# Influence of Hybridizing Flax and Hemp-Agave Fibers with Glass Fiber as Reinforcement in a Polyurethane Composite

**DOI:** 10.3390/ma9050390

**Published:** 2016-05-19

**Authors:** Pankaj Pandey, Dilpreet Bajwa, Chad Ulven, Sreekala Bajwa

**Affiliations:** 1 Department of Agricultural and Biosystems Engineering, North Dakota State University, Fargo, ND 58102, USA; pankaj.pandey@ndsu.edu; 2 Department of Mechanical Engineering, North Dakota State University, Fargo, ND 58102, USA; dilpreet.bajwa@ndsu.edu (D.B.); chad.ulven@ndsu.edu (C.U.)

**Keywords:** natural fiber composites, flax, hemp-agave, glass, polyurethane, hybridization

## Abstract

In this study, six combinations of flax, hemp, and glass fiber were investigated for a hybrid reinforcement system in a polyurethane (PU) composite. The natural fibers were combined with glass fibers in a PU composite in order to achieve a better mechanical reinforcement in the composite material. The effect of fiber hybridization in PU composites was evaluated through physical and mechanical properties such as water absorption (WA), specific gravity (SG), coefficient of linear thermal expansion (CLTE), flexural and compression properties, and hardness. The mechanical properties of hybridized samples showed mixed trends compared to the unhybridized samples, but hybridization with glass fiber reduced water absorption by 37% and 43% for flax and hemp-agave PU composites respectively.

## 1. Introduction

In the last few decades there has been a greater thrust toward curbing our reliance on non-renewable resources and finding viable options to decrease the burden on the environment. With increasing environmental regulations, the development of new materials that utilize a larger proportion of renewable and “green” raw materials has gained momentum. One such emerging area in the composites world is known as ‘biocomposites’ and it is finding its niche applications in building, automobile, and packaging industries [1]. Biocomposites contain one or more bio-based raw materials such as natural fibers. Natural fibers typically have high flexibility, low cost, ease of processing, biodegradability, and recyclability [2]. Several natural fibers such as coir [3], flax [4], hemp [5], bamboo [6], kenaf [7], abaca [8], and jute [9] have proven to be effective as reinforcements in composites. These biocomposites are typically used in non-structural applications because of their relatively low strength properties.

Polyurethanes (PUs) are a diverse class of polymers obtained from the reaction of polyols with isocyanates which give a highly crosslinked structure. They have successfully found applications in construction, automotive, and medical industries. The successful applications of PUs are credited to their ability to be produced as flexible to rigid structures [10]. The unique properties of PUs include their ease of processing, low viscosity, quick reaction time, no emission of volatile organic compounds, better economics, and excellent bonding with different fibers without the need of specific sizing of the fibers [11].

In spite of several advantages associated with the natural fibers, their inherent structure is a bottleneck to their successful application in polymer composites. While the natural fibers are polar and hydrophilic in nature because of the presence of hydroxyl groups in cellulose, the polymers are non-polar and hydrophobic in nature. This incompatibility severely affects the bonding between these two groups of materials. The poor adhesion between a natural fiber and a polymer matrix affects the mechanical properties of the material, and leads to high affinity to water and poor dimensional stability [12]. However, hybridization of different fibers in a composite can offer a balanced solution to enhancing the mechanical properties by utilizing other strong fibers such as glass fiber. A hybrid polymer composite usually utilizes two or more types of fibers with a single polymer matrix or a single type of fiber with two or more polymer matrices. The hybridization of fibers provides a unique opportunity to design a composite material in which the shortcomings in one fiber are compensated by the other fiber [13]. The improvement in water absorption properties in hybrid epoxy composites with jute and glass fiber reinforcement compared to jute-epoxy composites and glass-epoxy composites [14] is an example. Similarly, impact strength of the polyester composite was improved by hybridizing glass fiber with oil palm fiber, and with increasing the number of layers of glass fiber and oil palm fiber [15]. Also, the hybridization of composites with two natural fibers, such as silk/cotton and jute/cotton, has been shown to improve the tensile properties with increases in fiber content [16]

This paper presents a novel idea of combining natural fibers, such as flax and a hemp-agave mixture, with glass fiber in a polyurethane composite in order to improve the physico-mechanical properties of a natural fiber PU composite. To understand the impact of glass fiber hybridization with the natural fibers, both hybridized and unhybridized composite panels were manufactured and their physico-mechanical properties were compared.

## 2. Experimental Procedure

### 2.1. Fiber Mats

**Figure 1:**
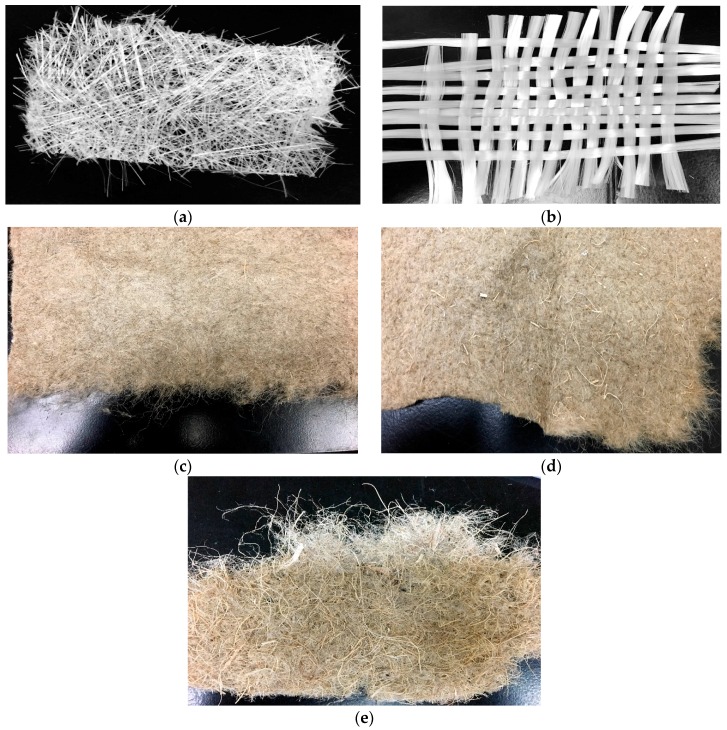
Different fiber mats used in the study. (**a**) non-woven glass mat; (**b**) woven glass mat; (**c**) flax1; (**d**) flax2; (**e**) hemp-agave.

The experiment involved manufacturing composite material samples with different types of fiber reinforcement. Different combinations of woven and non-woven glass fiber mats, flax fiber mats, and hemp-agave fiber mats were used as reinforcements. Fibers and fiber mats had distinctly different characteristics (Table 1). Although woven glass mat had the highest aerial density, the flax mats were much thicker which made them more difficult to penetrate (Figure 1).

### 2.2. PU Resin

The matrix plays a crucial role in transferring stress through interfacial layer to fiber, which is the main load bearing element during various applications. The matrix for the composite included a mixture of polyol resin, ELASTOPOR^®^P 12883R RESIN (BASF Corporation, Southfield, MI, USA) and a cross linking agent ELASTOPOR^®^P 1230 ISOCYANATE (BASF Corporation, Southfield, MI, USA), which yield a foamed based polymeric methylene diphenyl diisocyanate (MDI) based rigid polyurethane (PU) system with water as the sole blowing agent. The mix ratio of polyol to isocyanate used was 77 polyol/100 isocyanate measured by weight. The density of the mixture before foaming was 1.15 g/cm^3^.

### 2.3. Sample Preparation

**Figure 2:**
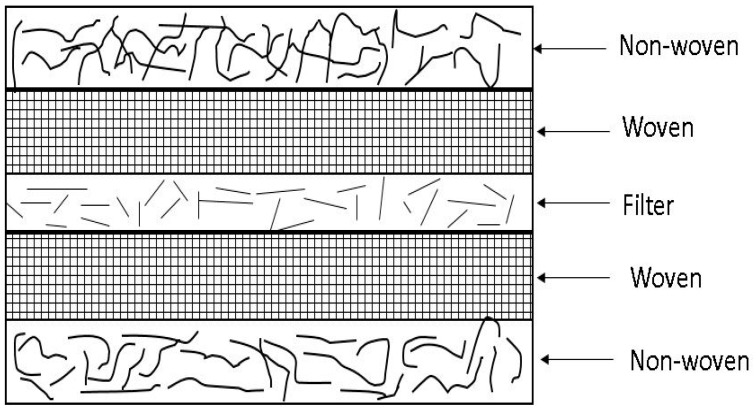
The hand lay-up of different layers of mats from bottom to top used in the polyurethane composite.

All the samples were made from PU resin and had an inner filter core made of glass in addition to the different types of fiber mats on both sides. The hybridized composite panels were made with two layers of fiber mats on each side of the filter as shown in Figure 2. In contrast, the unhybridized composite panels were constructed similarly to the hybridized composite panels but without the woven mat layers. The number of fiber mat layers, fiber orientation, and formulation can be adjusted to achieve specific thickness, density, and strength properties of the resulting composite material. These types of composites with glass as the woven and non-woven fiber reinforcement are currently available in the market for applications such as flooring and cabinetry.

The mass ratio of PU matrices varied from 62% to 77% between the different composites (Table 2). The mass ratio of the matrix was determined after preliminary trials were conducted to obtain complete coverage without any runoff of the PU resin. First, the mold was polished and then a mold-releasing agent was applied on the surface before placing the mat lay-up into it. Second, the mat layers of non-woven fiber, woven fiber, and filter were placed sequentially into the mold. Next, the resin and the cross linking agent for the PU matrix were mixed in a cup using a mechanical stirrer for ten seconds and then poured onto the filter layer. Finally, the top two layers of woven and non-woven fiber mats were quickly placed onto the PU matrix and covered by steel plates before the crosslinking reaction started to spread out rapidly. Note the whole arrangement of the mat lay-up was covered by a Teflon sheet (on the top and bottom) to prevent the panel from sticking to the steel plates. The mold was then placed on the lower movable platen of a hot press (Model 4122, Carver Inc., Wabash, IN, USA) set at 65 °C. The material was then pressed at 0.29 MPa pressure for 20 min. To ensure complete curing, the composite panels were post cured at room temperature for 15 min.

A total of six different set of samples were manufactured in the lab (Table 2) with different types of fiber reinforcement that resulted in different amounts of substrate. The proportion of fiber, resin, and cross linking agent used for each type of composite sample was optimized after preliminary trials aimed at obtaining evenly wet panels without any leak of PU resin outside the mold. Sample FG (flax1 and glass fiber) was made in a 203 × 203 × 19 mm^3^ mold because of the relatively poor penetrability of the flax fiber mat in 317.5 × 317.5 × 25.4 mm^3^ mold. Sample G’G’ (commercial glass fiber) was a commercially available material and was obtained in the size of 254 × 254 × 25.4 mm^3^.

### 2.4. Testing and Measurements

The samples manufactured in the lab were tested for various mechanical and physical properties following specific ASTM standards. The details of such properties tested are provided below:

#### 2.4.1. Void Content of Composites

The void content in all the composite samples was determined according to the ASTM D2734 [17]. The densities of the fibers and PU resin and their respective weight fractions used for different composite panels were used to determine the theoretical densities of the composite materials.

#### 2.4.2. Water Absorption Test

The water absorption of all composite samples was investigated in accordance with ASTM D570 [18]. Five samples per formulation were tested for percentage weight gain when submerged in water. The testing was performed on sample coupons of 76.2 mm length and 25.4 mm width. Composite samples were first dried in an oven at 50 °C for 24 h, and weighed to record the dry weight. The samples were then immersed in a water bath set at room temperature and the sample weight gain was recorded every 24 h for the duration of 27 days. The percentage weight gain each day was calculated as the incremental water absorption.

#### 2.4.3. Coefficient of Linear Thermal Expansion (CLTE)

The CLTE was measured for five samples from each formulation in accordance with ASTMD 6341 [19]. All the samples were conditioned sequentially, first in a freezer set at −23.4 °C, then at room temperature (23.5 °C), and finally in an oven set at 60 °C. For each of these three conditions, the samples were exposed for 48 h. At the end of each 48 h period, the length of the samples was recorded. The CLTE was measured as the slope of the curve of sample length *versus* temperature.

#### 2.4.4. Flexural Properties

The flexural properties of the samples were tested according to the ASTM D790 [20], which is a three point flexural test method for unreinforced and reinforced plastics. Due to the limitations on the mold size, a span-to-depth ratio different than the 16:1 recommended in the standard was used to determine modulus of elasticity (MOE) and modulus of rupture (MOR). Instead, a span-to-depth ratio of 10.5:1 was used for samples GG (Glass Fiber), HG (Hemp-Agave and Glass Fiber), F (Flax2), and H (Only Hemp-Agave Fiber). For the commercial panels G’G’ (Commercial Glass Fiber), and flax1 samples, an 8:1 and 6.4:1 span-to-depth ratio were used, respectively, because of their smaller sample sizes. The cross head motion rate was calculated according to the span length, width, and thickness of the samples as specified in the standard. A universal testing machine (312 Frame, Test Resources, Shakopee, MN, USA) was used for testing the materials.

#### 2.4.5. Compression Properties

All the samples were tested for compression properties in accordance with ASTM D695 [21]. All the specimens were cut as specified in the standard. The cross head motion rate of 1.3 mm/min was used. The compressive strength and modulus of elasticity were calculated from the stress-strain data recorded by the universal testing machine (312 Frame, Test Resources, Shakopee, MN, USA).

#### 2.4.6. Hardness

The surface hardness of the samples was tested in accordance with ASTM D1037 [22] using the universal testing machine (312 Frame, Test Resources, Shakopee, MN, USA). The modified Janka-ball test method used a ball with an 11.3 mm in diameter. The specimen size of 76.2 × 152.4 × 25.4 mm^3^ was penetrated by the “ball” until it reached half of its diameter and the load value was recorded at that point. Two penetrations each were made on both sides on the flat faces of the panel. A cross head motion rate of 6 mm/min was used.

### 2.5. Light Microscope Images

The samples tested during flexural bending were analyzed using stereo light microscope SZM 7045 (AmScope, Irvine, CA, USA) to study their morphology. The images of the fracture surface of the samples were collected at a magnification of 25×.

### 2.6. Statistical Analysis

In order to establish a comparison of means between treatments, ANOVA tests were performed. Two sample *t*-tests were performed between all formulations to compare treatment means for the various properties tested. Both ANOVA and two sample t-tests were performed with the software Minitab 17 (Minitab Inc., Pennsylvania State University, Central County, PA, USA). The error bar in the graphs is the standard deviation of the mean for the dataset. The strength properties obtained by hardness, flexural, and compression tests were first normalized by the respective panel densities before doing statistical analysis as material density can affect strength properties of the material, and different formulations showed large variability in material density.

## 3. Results and Discussion

The sample manufacturing process was difficult for the samples containing flax or hemp-agave mat. The natural fiber mats interfered with the penetration and distribution of the PU resin. Further, the resin-crosslinking mix did not spread uniformly to wet and encapsulate the fibers, particularly in large molds, leaving the outermost layers of the natural fibers partially exposed. This was one of the reasons for using the smaller molds. Additionally, flax and hemp-agave mats were not available in preferred specification including lower density and thickness. Although flax is grown locally for grain and the crop residue left in the field is a good source of strong fibers, there are no flax fiber processing industries in the US.

### 3.1. Void Content

The presence of voids in a composite material leads to a reduction in its mechanical and physical properties. The formation of voids can be attributed to the trapped air or other volatiles present in the composite materials [23]. The void content (%) of composite samples made with different combinations of fibers is presented in Table 3. The highest amount of void (7.9%) was exhibited by the flax alone composite (F) showing the incompatibility between the PU resin and the flax2 mat. However, when the flax1 mat was hybridized with the glass fiber mat, a void content of 4.8% was observed, which depicts a better interaction of the flax1 fiber mat with the PU resin than the flax2 mat. The lowest void content of 4.1% was observed for the hemp alone (H) composite which increased to 4.8% when hybridized with the glass fiber (HG). This increase in void content shows that when the hemp fiber mat was used as the outermost layer, the resin couldn’t interact with the hemp skin layers as well as it did in the hemp alone samples. The void content in the glass fiber composite (GG) was 4.8% whereas the commercial glass fiber composites (G’G’) showed only 1.4% void content.

### 3.2. Water Absorption

**Figure 3:**
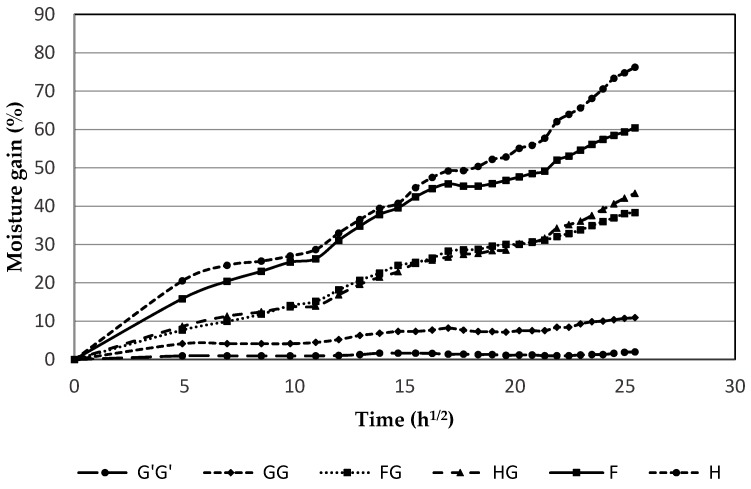
Water absorption of composite samples at room temperature plotted against square root of time for composite samples containing commercial sample with glass fiber (G’G’), glass fiber (GG), flax and glass fiber (FG), hemp and glass fiber (HG), flax alone (F), and hemp alone (H).

Water absorption in composites is important to study as it may cause debonding, disruption of integral structure, and loss of strength [24], particularly in applications where the material may be exposed to water. All composite samples containing natural fibers showed much higher water absorption than those containing only glass fibers throughout the data collection period (Figure 3). The initial rate of water absorption was higher for unhybridized natural fiber composite samples. Hemp composite samples (H) showed the highest moisture gain of 76%, followed by flax composite samples (F) with 60% moisture gain. The natural fibers are inherently hydrophilic and using the flax and hemp fibers as the skin layers made them highly susceptible to the moisture absorption. Natural fibers swell when they absorb water, which can lead to micro-cracking of the material, further hampering the integrity of the interfacial surface. The swelling stresses and dimensional instability of the composite when the natural fiber absorbs water will subsequently lead to failure of the composites [25]. When hybridized with glass fiber, a drastic drop of 43% and 37% in moisture absorption was observed for composites containing hemp-agave and flax, respectively. This drop in moisture uptake was expected because of the high resistance of glass fibers to moisture. The lowest moisture absorption of 12% and 2% were observed in unhybridized glass fiber composite samples of GG and G’G’, respectively.

### 3.3. Coefficient of Linear Thermal Expansion (CLTE)

**Figure 4:**
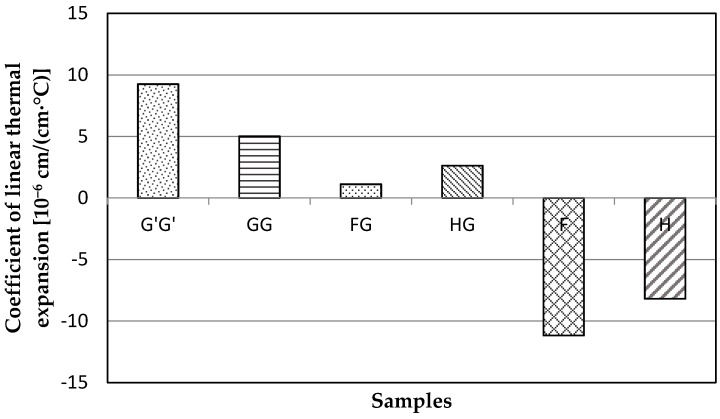
Coefficient of thermal linear expansion for composite samples with commercial glass fiber alone (G’G’), glass fiber (GG), flax and glass fiber (FG), hemp-agave and glass fiber (HG), flax fiber alone (F), and hemp fiber alone (H) measured with ASTM D6341.

Composite samples containing only natural fiber reinforcements such as flax and hemp fibers showed negative CLTE while all other samples showed positive values (Figure 4). This is expected as natural fibers are expected to shrink under elevated temperature while glass fibers are expected to expand. The highest absolute value of CLTE was observed for samples containing only glass fibers (G’G’) and samples containing only flax fiber reinforcement (F). It is notable that the hybrid composites showed the lowest CLTE, which is preferred in applications such as flooring and cabinetry, or where the material will be exposed by large temperature fluctuations. A low CLTE would ensure high dimensional stability when exposed to temperature extremes.

### 3.4. Flexural Properties

**Figure 5:**
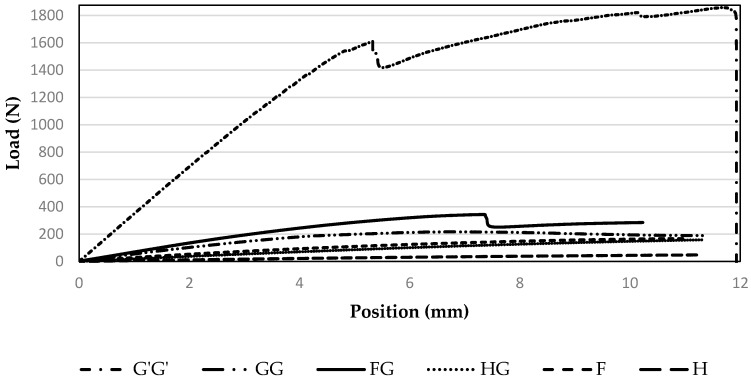
Flexural load-position curves of the composites containing commercial glass fiber (G’G’), glass fiber (GG), flax and glass fiber (FG), hemp and glass fiber (HG), flax alone (F) and hemp alone (H).

During the flexural test, no sample broke under the recommended strain value of 5%. The load *vs*. position curve depicts the change in the load value with change in the position of the composites (Figure 5). It can be observed that the commercial glass samples behaved much better than other samples under the flexural load. Initially, flax hybridized samples behaved well but this trend didn’t transcend into higher specific flexural properties than glass fiber based composite samples (GG).

**Figure 6:**
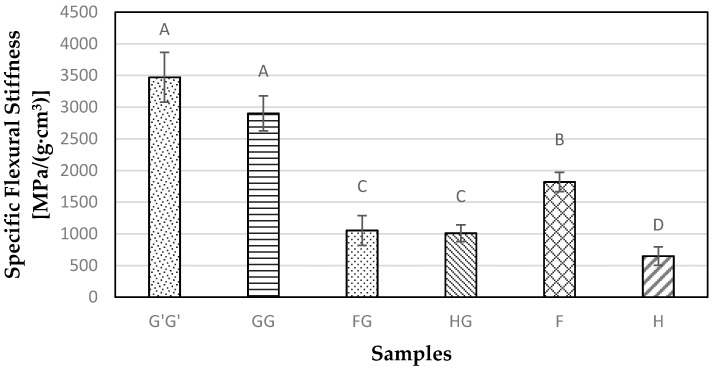
Specific flexural stiffness results for composite samples containing commercial Glass fiber alone (G’G’), Glass fiber alone (GG), Flax and Glass fiber (FG), Hemp-agave and Glass fibers (HG), Flax fiber alone (F), and Hemp fiber alone (H).

The flexural MOE and MOR values were calculated as specified by the ASTM D790 standard, which were further normalized by the respective densities of the panels to eliminate the effect of density across all the formulations. Both types of glass fiber reinforced unhybridized composite samples (G’G’ and GG) exhibited the highest specific flexural modulus compared to both natural fiber reinforced samples (Figure 6). The formation of an interphase region between a glass fiber and a PU matrix allows for the better dispersion and proper wetting of fibers into the matrix leading to better mechanical properties of the composites [26]. The addition of glass fiber resulted in lowering of modulus in hybridized flax composite samples by 42%, whereas the hybridization of hemp fiber resulted in a 55% improvement in modulus (*p* < 0.05), when compared with just hemp fiber reinforced composite samples.

**Figure 7:**
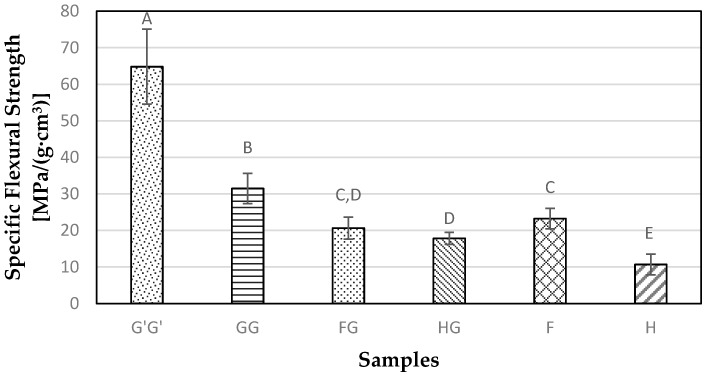
Specific flexural strength for composite samples containing commercial Glass fiber alone (G’G’), Glass fiber alone (GG), Flax and Glass fiber (FG), Hemp-agave and Glass fibers (HG), Flax fiber alone (F), and Hemp fiber alone (H).

The highest specific flexural strength (flexural strength normalized by material density) of 64.81 MPa/g/cm^3^ was observed for G’G’ samples (Figure 7). The GG samples showed a decrease of 51% in specific flexural strength compared to G’G’ samples. The high strength of G’G’ samples may be credited to better manufacturing processes, such as even mixing and distribution of resin and cross linking agent, and better encapsulation of fibers in the commercial sample. All natural fiber reinforced composite samples showed significantly lower (*p* < 0.05) specific flexural strength than G’G’ and GG samples. There was an improvement of 67% in specific flexural strength observed for hybridized hemp samples whereas hybrid flax samples did not result in significant change (*p* > 0.05) for specific flexural strength.

**Figure 8:**
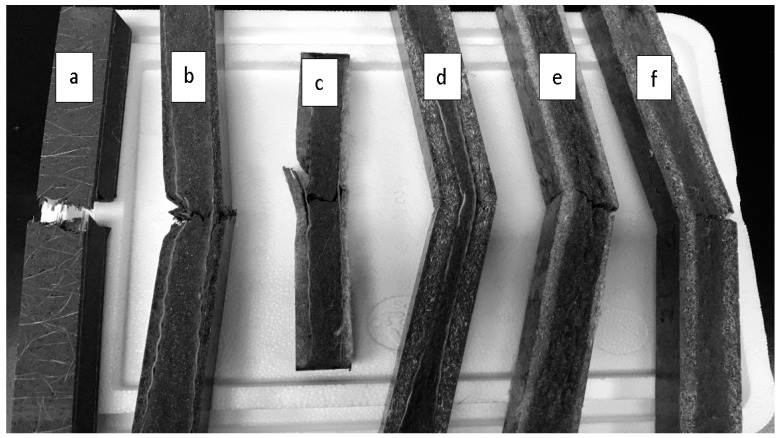
Images of composite samples tested for flexural properties showing the failure surface. (**a**) commercial glass (G’G); (**b**) glass (GG); (**c**) hybrid flax and glass (FG); (**d**) hybrid hemp and glass (HG); (**e**) only flax (F); and (**f**) only hemp (H).

The flexural fracture behavior of samples depends mainly on the tensile strength of the material as the initiation of cracking always occur on the tension side, which then travels across the width to the compression side until the sample fails. To understand the fracture behavior, all the samples were flexurally loaded until they failed. Of all the fiber reinforced samples tested, only the commercial glass fiber reinforced sample broke apart completely (Figure 8).

**Figure 9:**
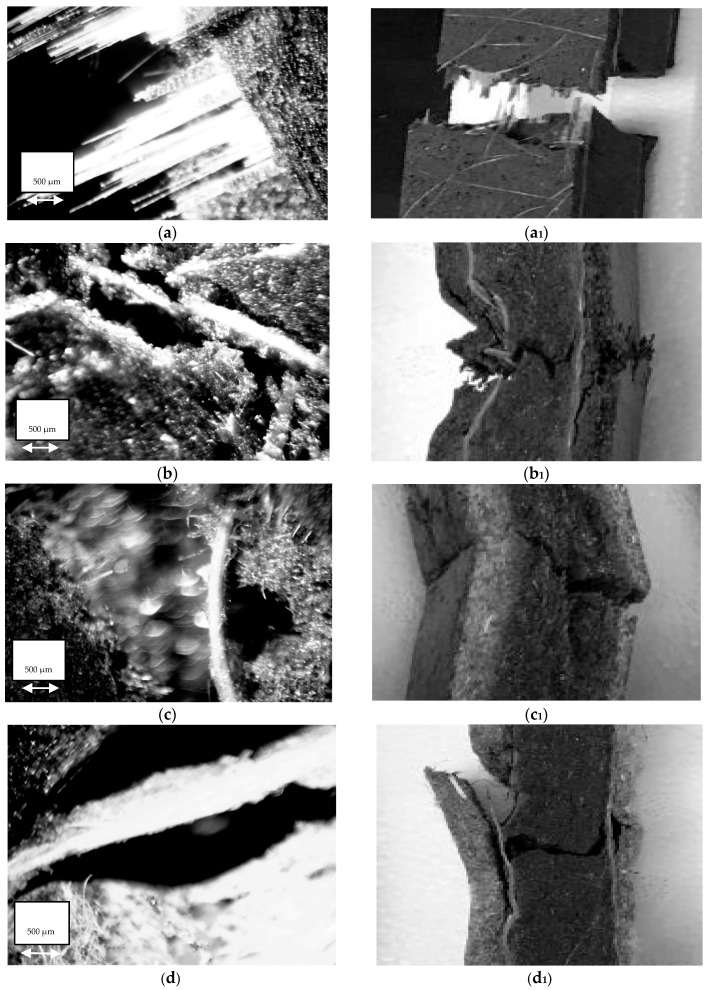

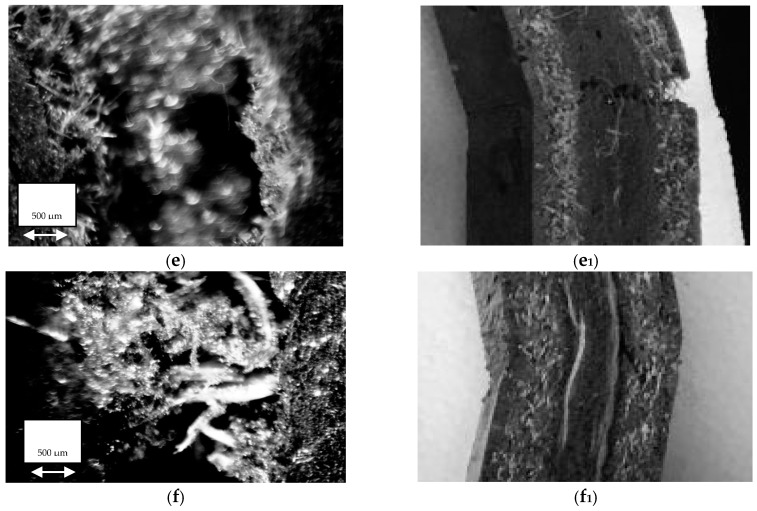
Light microscopy images captured from the tension side of all the flexurally broken samples. (**a**,**a_1_**) commercial glass (G’G’); (**b**,**b_1_**) glass (GG); (**c**,**c_1_**) only flax (F); (**d**,**d_1_**) hybrid flax and glass (FG); (**e**,**e_1_**) only hemp (H); and (**f**,**f_1_**) hybrid hemp and glass (HG).

In the commercial samples, the outermost non-woven glass fiber layer on the tension side was well dispersed in the PU matrix as no fiber pull out was observed (Figure 9a,a_1_). When these samples were flexurally loaded, the outermost non-woven glass fiber bears the load up to its maximum capacity and then transfers this load to the woven glass fiber. However, the woven glass fiber was found to be poorly mixed with the matrix which was apparent from the clean long glass fibers pulled out with no adherence to the matrix. On the other hand, the glass reinforced samples manufactured in our lab did not break completely though all the commercial samples did. Also, the mechanism of crack propagation in GG samples observed was different than in the commercial samples. The crack initiated from the tension side and the outermost layer of non-woven glass fiber failed after reaching its maximum capacity. The crack propagated through the non-woven glass fiber to the next interface of woven glass and the PU matrix (Figure 9b,b_1_). The woven glass fiber was found to be strong enough to prevent the crack from transcending through it; rather, the crack was found to be running along the surface due to lack of bonding between the two layers. During this time, the core of the glass filter, being weaker in strength than woven glass fiber, initiated a crack due to the continuous increasing load from the compression side. The failure of the sample was then continued from the core towards the compression side.

The crack propagation during flexural bending of flax and hemp fiber and their hybridized samples is shown in Figure 9c–f,c_1_–f_1_. The crack initiated in all of these samples on the tension side. In flax fiber reinforced samples (F), the fiber pull out from the outermost layer was the origin of the failure mode. Non-woven natural fiber mats consisted of fibers of different lengths which can explain the bigger fiber pull outs in the image. For flax and glass fiber reinforced samples (FG), a change in direction of the propagation of crack was observed with the increase in load value as was observed in GG samples. The crack initiation started on the tension side but the flax fiber having high extensibility did not allow propagation through it to the stronger woven glass fiber. The crack appears to run along the flax and woven glass fiber layer, and as the load kept on increasing from the compression side, the crack again initiates but from the glass filter core (it being weaker than woven glass fiber). The crack then propagated from the core upwards to the woven glass fiber and woven layers, and failure of these upper layers of fibers appear. The same mechanism of crack propagation appears during hemp and its hybridized samples. The hemp fiber pull out from matrix was the main reason for the failure of hemp reinforced samples. The crack initiated at the tension side and propagated through the core layer of the glass filter with no change in propagation direction. However, when hybridized with glass fiber, the crack initiation started from the tension side but it could not propagate in the same direction as it encountered the woven glass fiber layer. The crack appeared to change its direction and started propagating along the interface between woven glass fiber and hemp fiber. 

### 3.5. Compressive Properties

**Figure 10:**
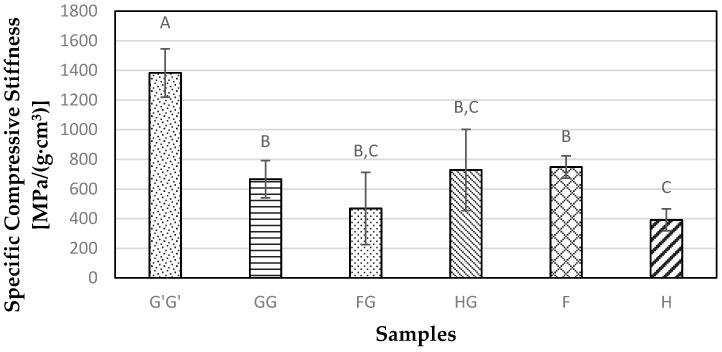
Specific **c**ompressive stiffness for composite samples containing commercial Glass fiber alone (G’G’), Glass fiber alone (GG), Flax and Glass fiber (FG), Hemp-agave and Glass fibers (HG), Flax fiber alone (F), and Hemp fiber alone (H).

**Figure 11:**
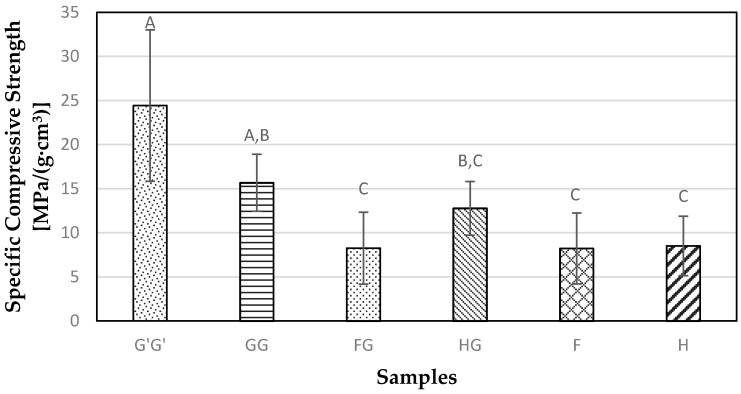
Measure of specific compressive strength obtained for composite samples containing commercial Glass fiber alone (G’G’), Glass fiber alone (GG), Flax and Glass fiber (FG), Hemp-agave and Glass fibers (HG), Flax fiber alone (F), and Hemp fiber alone (H).

The highest specific compressive modulus value observed was 1382.82 MPa/(g·cm^3^) for the G’G’ sample. On the other hand, the specific compressive modulus for GG samples was not statistically different (*p* > 0.05) from FG, HG, and F samples (Figure 10). The glass fiber in the FG sample has a higher modulus and strength than the flax fiber, but when the tension force goes beyond a certain point the glass fiber breaks first since it has lesser extensibility than the natural fibers. This leads to a stress concentration resulting in a failure of stress transfer to the flax fiber causing the composites to fail. Figure 11 shows the results from the specific compressive strength of all the composite samples tested in the study. The highest compressive strength was observed for G’G’ samples at 24.43 MPa/(g·cm^3^). There was no significant difference (*p* > 0.05) observed between natural fiber reinforced samples.

### 3.6. Hardness Testing

**Figure 12:**
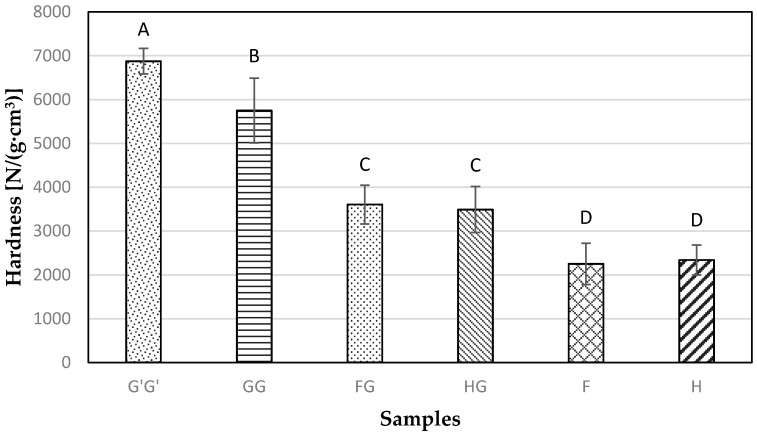
Specific hardness testing results obtained for composite samples containing commercial Glass fiber alone (G’G’), Glass fiber alone (GG), Flax and Glass fiber (FG), Hemp-agave and Glass fibers (HG), Flax fiber alone (F), and Hemp fiber alone (H) using Janka ball.

The commercial glass based samples G’G’ showed the highest specific hardness value of 6875.46 N/(g·cm^3^), followed next by GG samples (Figure 12). The unhybridized flax and hemp-agave samples showed no significant (*p* > 0.05) difference between each other with the lowest value for hardness among all the samples tested. When hybridized, both flax and hemp-agave samples showed a significant increase (*p* < 0.05) in the hardness property due to the better resistance provided by the glass reinforcement.

## 4. Conclusions

This study explored the viability of using natural fiber in combination with synthetic glass fiber in a hybrid PU composite. The hybridization of flax and hemp-agave fibers with glass fiber in polyurethane composite was performed to investigate their physical and mechanical properties. The void content of 7.9% was observed for flax2 mat reinforced samples, showing its incompatibility with the PU resin. The hybrid samples from flax and hemp-agave fibers showed considerable improvement in water absorption properties to the unhybridized flax and hemp composite samples by 37% and 43%, respectively. The specific flexural properties showed that the hybridized flax and hemp-agave composite samples behaved similarly while the flax alone composite showed better specific flexural stiffness than all other combinations of flax and hemp-agave composite samples. The specific flexural strength results showed no significant difference between the flax and hemp-agave hybridized composite samples. The compressive tests showed no improvements among all the natural fiber reinforced samples, even after hybridizing with the glass fiber. Hybridization of flax and hemp-agave fiber composites improved their hardness properties because of the addition of the high strength glass fibers. 

## Figures and Tables

**Table 1:** Characteristics of the fiber mats used in the study. Fiber length and thickness are measured for 15 fibers from each mat.

Fiber Mats	Type	Average Fiber Length (mm)	Average Fiber Diameter (µm)	Arial Weight of Mat (g/m^2^)	Mat Thickness (mm)	Source
Glass	Non-woven (spunlaid)	37.08	40	450	1.25	SpaceAge Synthetics, Fargo, ND, USA
Glass	Woven (bi-axial)	304.80	100	600	1.50	SpaceAge Synthetics, Fargo, ND, USA
Flax1	Non-woven (needle punched)	63.25	20	500	4.00	Hemp-Flax, Groningen, Netherlands
Flax2	Non-woven (needle punched)	77.56	40	550	5.50	Composite Innovation Centre, Winnipeg, MB, Canada
Hemp-Agave	Non-woven (needle punched)	71.51	200	352	5.40	Composite Innovation Centre, Winnipeg, MB, Canada

**Table 2:** Composition of the fiber reinforced composite material samples manufactured in the lab. Commercial Glass Fiber (G’G’); Glass Fiber (GG); Flax1 and Glass Fiber (FG); Hemp-Agave and Glass Fiber (HG); Only Flax2 Fiber (F); Only Hemp-Agave Fiber (H).

Sample Name	Non-Woven Mat	Woven Mat	Filter	PU Resin (Weight %)	Mold Size (mm^3^)	Panel Density (g/cm^3^)
G’G’	Glass	Glass	Glass	67–70	254 × 254 × 25.4	0.45
GG	Glass	Glass	Glass	65	317.5 × 317.5 × 25.4	0.22
FG	Flax1	Glass	Glass	65	203.2 × 203.2 × 19.1	0.33
HG	Hemp-Agave	Glass	Glass	62	317.5 × 317.5 × 25.4	0.22
F	Flax2	-	Glass	77	317.5 × 317.5 × 25.4	0.20
H	Hemp-Agave	-	Glass	77	317.5 × 317.5 × 25.4	0.16

**Table 3:** The void content of flax, hemp-agave, and glass fiber reinforced composites.

Type of Composites	Void Content (%)
Commercial Glass Fiber (G’G’)	1.4
Glass Fiber (GG)	4.8
Flax1 and Glass Fiber (FG)	4.8
Hemp-Agave and Glass Fiber (HG)	5.2
Only Flax2 Fiber (F)	7.9
Only Hemp-Agave Fiber (H)	4.1
